# Safe harboring and governance: Lakota relational systems as context for women's health integration

**DOI:** 10.3389/fsoc.2026.1793764

**Published:** 2026-04-13

**Authors:** Lawrence Merle Nelson

**Affiliations:** Mary Elizabeth Conover Foundation, Inc., McLean, VA, United States

**Keywords:** care fragmentation, complexity in health systems, continuity of care, health systems governance, Primary Ovarian Insufficiency, relational governance, trust and legitimacy, women's health integration

## Abstract

This Perspective develops a sociological model of *safe harboring* to explain how governance design shapes the capacity of health systems to sustain integrated care. Drawing on long-standing relational familiarity with Lakota governance practices, articulated here as the “Sundance Ecosystem,” the analysis examines women's health—and Primary Ovarian Insufficiency in particular—as a structural stress test for contemporary health systems. Women's health routinely spans multiple physiological systems, social roles, and time horizons, exposing limitations in governance arrangements organized around administrative silos and episodic coordination. The Sundance Ecosystem is treated as an orienting relational governance model rather than as an object of study or empirical case. It informs the articulation of *safe harboring* as a governance condition defined by continuity, distributed responsibility, and legitimacy grounded in sustained participation rather than hierarchical control. This orientation complements, rather than replaces, sociological theory by clarifying governance features often obscured within institution-centered systems. The manuscript argues that fragmentation in women's health research persists not as a technical failure of coordination but as a structural consequence of governance design. *Safe harboring* reframes integration as a governance property, emphasizing the conditions under which health systems can sustain continuity, relational accountability, and interpretive coherence across complex, intersecting health needs. This Perspective articulates these concepts for the governance of Primary Ovarian Insufficiency care and human investigation, demonstrating how continuity, responsibility, and interpretive integration can be structurally supported across both clinical and research domains.

## Introduction

Health systems are often evaluated through efficiency, outputs, and technical coordination. Sociological scholarship, however, has long emphasized that systems of care are also moral and relational systems shaped by governance design, in which trust, legitimacy, and accountability emerge from structured relationships rather than technical performance alone (Kleinman, 1978; Gilson, 2025; Peters, 2024). From this perspective, the organization of authority, responsibility, and communication within institutions is not secondary to care delivery but constitutive of how care is experienced and how outcomes are produced.

Persistent calls for integration in health research have largely focused on improving coordination across disciplines and conditions, particularly through cross-sector and policy alignment (Saikat et al., 2025; Haby et al., 2025). Yet fragmentation remains a defining feature of contemporary health systems, with well-documented consequences for care continuity, coordination, and patient outcomes (Haggerty et al., 2003; Kern et al., 2024). This persistence suggests that fragmentation is not simply a technical failure of coordination but a structural outcome of governance arrangements that distribute knowledge, authority, and responsibility across institutional boundaries (Saikat et al., 2025; Plsek and Greenhalgh, 2001). Efforts to improve integration that do not address these underlying governance conditions may therefore have limited impact.

Women's health provides a particularly revealing analytic lens because it routinely spans multiple physiological systems, social roles, and time horizons simultaneously. Many conditions require longitudinal interpretation, coordination across specialties, and sustained engagement between clinicians and patients—features that are difficult to maintain within systems organized around episodic encounters and administrative silos. Primary Ovarian Insufficiency exemplifies these challenges, as it unfolds across reproductive, endocrine, cardiovascular, and psychosocial domains and requires continuity of care for effective management (Nelson, 2009; Pereira Gray et al., 2018). When such continuity is disrupted, the interpretive work required for diagnosis and management becomes fragmented, and responsibility for care may become diffuse across providers and institutions.

**Figure 1:**
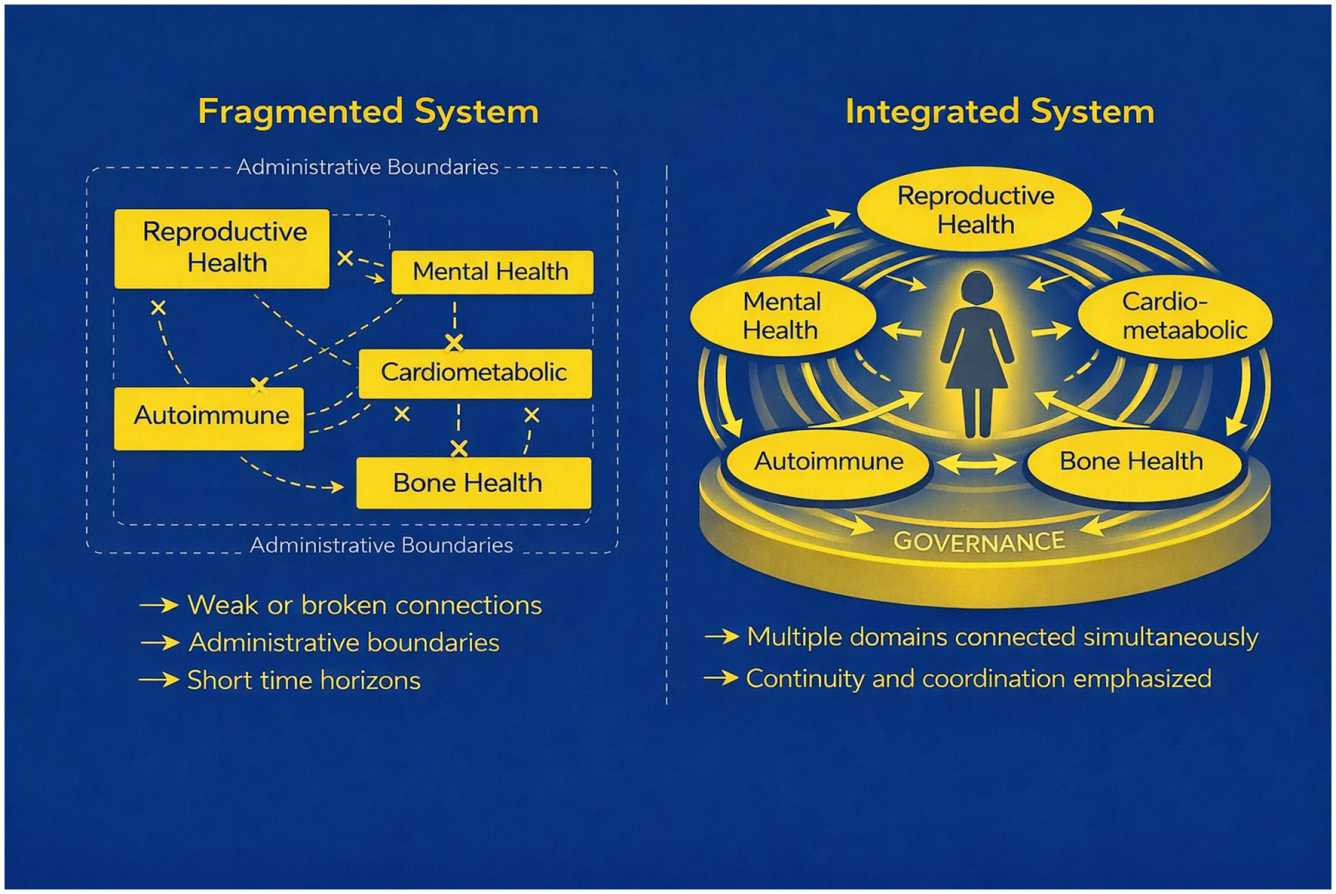
Fragmentation vs. integration in women's health research. This figure contrasts fragmented and integrated approaches to women's health research and care. Fragmented systems are organized around administrative and disciplinary boundaries, with weak or broken connections across domains and short time horizons. Integrated systems coordinate multiple health domains simultaneously around the individual, emphasizing continuity, relational connection, and governance structures that enable sustained integration over time.

Figure 1 illustrates the contrast between fragmented and integrated approaches to women's health research and care. In fragmented systems, knowledge, responsibility, and decision-making are distributed across disconnected domains, limiting the capacity for cumulative understanding and coordinated response. In integrated systems, governance arrangements support continuity, relational coordination, and shared interpretation across time. This contrast establishes the central problem addressed in this article: whether health systems can sustain the relational and interpretive conditions necessary for integration, or whether fragmentation persists as a structural feature of governance design.

## Methodological framing, boundary conditions, and the Sundance Ecosystem as an orienting governance model

This manuscript is informed by a reflexive background orientation and sociological theory. The Sundance Ecosystem is treated as an orienting relational governance model that informed the articulation of a *safe harboring* framework, rather than as an object of study, data source, or empirical case. This approach is consistent with sociological traditions that use reflexive positioning to clarify analytic perspective while grounding interpretation in established theoretical frameworks of health systems as relational and institutional arrangements (Kleinman, 1978; Gilson, 2025). Within this framing, reflexivity functions not as a substitute for analysis but as a means of making explicit how governance assumptions are identified and interpreted. No clinical research, intervention, or data collection was conducted within Indigenous communities.

The analytic work of this article remains grounded in medical sociology and health systems scholarship concerning legitimacy, trust, institutional distance, and governance design. Contemporary research demonstrates that trust and legitimacy in health systems are not solely interpersonal phenomena but are produced through governance arrangements that structure accountability, responsiveness, and participation (Kalulu et al., 2025; Peters, 2024). Similarly, institutional design shapes how individuals engage with health systems and how trust is sustained or eroded across broader social and political contexts (Menon et al., 2025). These literatures provide the primary analytic foundation for the arguments developed in this manuscript and situate *safe harboring* as a governance construct rather than a cultural or normative claim.

References to Lakota relational systems are included to clarify ethical posture and analytic orientation rather than to serve as empirical evidence or generalizable cultural description. Indigenous scholarship has emphasized that health and wellbeing are embedded within relational systems that integrate community, history, and responsibility across time (Kirmayer et al., 2011; Gone, 2013; Redvers et al., 2024). In this manuscript, such perspectives serve as an orienting contrast, sharpening attention to governance features—particularly continuity, distributed responsibility, and relational legitimacy—that may be obscured within institution-centered systems. These references are not offered as comprehensive representations of Lakota knowledge systems nor as substitutes for sociological theory, but as a bounded interpretive lens that informs the development of the safe harboring construct.

Within this orienting frame, the Sundance (Wi Wanyang Wacipi) in the Lakota tradition can be understood as a relational governance system centered on continuity, renewal, and distributed responsibility. It is not a single event but a sustained communal commitment involving preparation, kinship obligation, moral accountability, and leadership exercised through elders and ceremonial leaders (Walker, 1982; Powers, 1977). Participation is structured through relational roles rather than hierarchical control, and responsibility is distributed across individuals, families, and community members over time. The Sundance affirms that individual wellbeing is inseparable from family, community, land, and shared responsibility, reflecting a relational ontology in which governance, health, and meaning are interwoven rather than separated domains (Deloria, 1994; Redvers et al., 2024).

For readers unfamiliar with the Sundance, it is a multi-day communal gathering that includes preparation, ceremony, and sustained participation by individuals and families. Participants often engage in periods of fasting, prayer, and physical endurance, while the broader community supports the process through shared responsibilities such as organizing, providing food, maintaining the ceremonial space, and caring for one another (Powers, 1977; Walker, 1982). The ceremony is structured around a central gathering place, often symbolically organized, where participants come together in a coordinated and purposeful way under the guidance of ceremonial leaders and elders. Activities unfold over time rather than as a single event, with meaning emerging through collective participation, commitment, and continuity. This description is offered solely to orient readers to the form and communal nature of the Sundance, without attempting to represent its full cultural, spiritual, or ceremonial significance.

As described by Peter Catches, the sacred fireplace serves as a relational center that holds continuity, accountability, and collective orientation rather than authority or control (Catches, 1999). Within this configuration, coherence is maintained through relational anchoring rather than hierarchical oversight. Roles are distributed, responsibility is sustained across time, and legitimacy emerges through participation rather than formal authority. These features resonate with broader sociological accounts of relational systems in which trust and coherence arise from sustained engagement rather than formal control structures (Kirmayer et al., 2011; Gone, 2013).

In this manuscript, the Sundance is not presented as an object of study or as a transferable Indigenous system. Rather, familiarity with its relational structure informs the articulation of *safe harboring* as a governance condition characterized by continuity, distributed responsibility, and relational legitimacy. Its relevance lies in clarifying structural features of governance that may be obscured within institution-centered health systems. This contrast clarifies the central claim of this article: that fragmentation in contemporary health systems is not an inevitable feature of complexity, but a consequence of governance arrangements that fail to sustain relational continuity, distributed responsibility, and interpretive coherence across domains.

## Fragmentation and integration in women's health research

Fragmentation in women's health research reflects institutional organization rather than the intrinsic nature of women's health itself, and therefore must be understood as a structural outcome of governance design rather than a failure of coordination. Sociological and governance scholarship has long shown that health systems are structured through administrative, disciplinary, and policy boundaries that shape how problems are defined, studied, and managed (Plsek and Greenhalgh, 2001; Saikat et al., 2025). Within such systems, research domains are frequently organized as discrete problems, limiting continuity, constraining cumulative understanding, and separating forms of knowledge that must be integrated in practice. This fragmentation is therefore not simply a failure of coordination but a structural outcome of how authority, expertise, and accountability are distributed within health systems.

Women's health provides a particularly revealing case because it routinely spans multiple physiological systems, life stages, and social contexts. Conditions such as Primary Ovarian Insufficiency require longitudinal interpretation across endocrine, reproductive, cardiovascular, and psychosocial domains, yet research and care pathways are often organized within narrower specialties and short-term analytic frames (Nelson, 2009; Haggerty et al., 2003). Empirical studies of care fragmentation demonstrate that such structural separation is associated with gaps in coordination, reduced continuity, and increased risk of adverse outcomes (Kern et al., 2024). At the same time, continuity of care has been consistently associated with improved outcomes, including reduced mortality, highlighting the importance of sustained relational and informational integration across time (Pereira Gray et al., 2018).

Fragmentation operates across multiple dimensions. *Institutional fragmentation* arises when administrative and disciplinary silos separate domains of expertise. *Temporal fragmentation* occurs when systems are organized around episodic encounters rather than longitudinal care relationships. *Epistemic fragmentation* emerges when different forms of knowledge—clinical, experiential, and social—are generated and interpreted in isolation rather than in relation to one another. Governance-oriented reviews of public health systems highlight how sectoral boundaries and misaligned incentives reinforce fragmentation, undermining integration across complex health needs (Saikat et al., 2025; Haby et al., 2025).

As illustrated in Figure 1, fragmented systems are characterized by administrative separation, weak inter-domain connections, and short time horizons. These systems often rely on coordination across boundaries rather than continuity within relationships, placing increasing interpretive and logistical burdens on patients and clinicians navigating care (Kyle et al., 2025). Digital infrastructures further shape these dynamics, as electronic health systems can introduce tensions between administrative standardization and clinical interpretation, sometimes producing unintended consequences for care coordination and patient safety (Greenhalgh et al., 2009; Abdelaziz et al., 2024). Integrated systems, by contrast, coordinate multiple domains simultaneously and emphasize continuity across time, allowing clinical signals, patient priorities, and evolving evidence to be interpreted within a coherent relational context. Integration does not eliminate specialization; rather, it requires governance arrangements capable of sustaining relational coordination, preserving identifiable responsibility, and maintaining feedback across domains within complex health systems.

## Governance design and safe harboring

The term “*safe harboring*” is used here as a sociological descriptor rather than a metaphorical or romantic label. It denotes governance arrangements that structurally enable continuity, relational legitimacy, and sustained participation across time. In this sense, *safe harboring* is analytically aligned with established constructs in the governance and trust literature, which emphasize that legitimacy and trust emerge from institutional arrangements that maintain accountability, responsiveness, and meaningful engagement with those affected by decisions (Kalulu et al., 2025; Peters, 2024; Menon et al., 2025). Rather than treating trust as an outcome to be engineered through communication or policy, this framework locates trust as a property of governance design. In this sense, *safe harboring* specifies the governance conditions under which continuity, accountability, and relational legitimacy can be sustained within complex health systems, emphasizing that integration depends less on coordination mechanisms than on how responsibility, participation, and continuity are structured over time.

Institution-centered governance models typically prioritize hierarchy, standardization, and accountability at scale. These features can support coordination across large and complex systems; however, sociological and health systems research demonstrates that they may also increase abstraction from clinical experience, particularly when decision authority becomes distant from the contexts in which care is delivered (Gilson, 2025; Haby et al., 2025). Studies of electronic health infrastructures further demonstrate that system design can reshape clinical work in ways that are not always visible to governance structures, introducing unintended consequences for safety, coordination, and accountability (Greenhalgh et al., 2009; Abdelaziz et al., 2024). In such systems, responsibility may become diffuse, feedback may be attenuated, and participation may be constrained by formal structures, contributing to gaps between institutional intent and experiential reality.

In contrast, the *safe harboring* governance model emphasizes layered relational embedding, distributed responsibility, and continuity across time. Governance is organized not solely through hierarchical control but through sustained relationships that preserve interpretive context and accountability. This orientation is consistent with research demonstrating that continuity of care supports coherent interpretation, coordination, and improved outcomes in complex health conditions (Haggerty et al., 2003; Pereira Gray et al., 2018). It also reflects insights from complexity science, which highlight the importance of feedback, adaptation, and distributed decision-making in maintaining system coherence within dynamic environments (Plsek and Greenhalgh, 2001).

**Figure 2:**
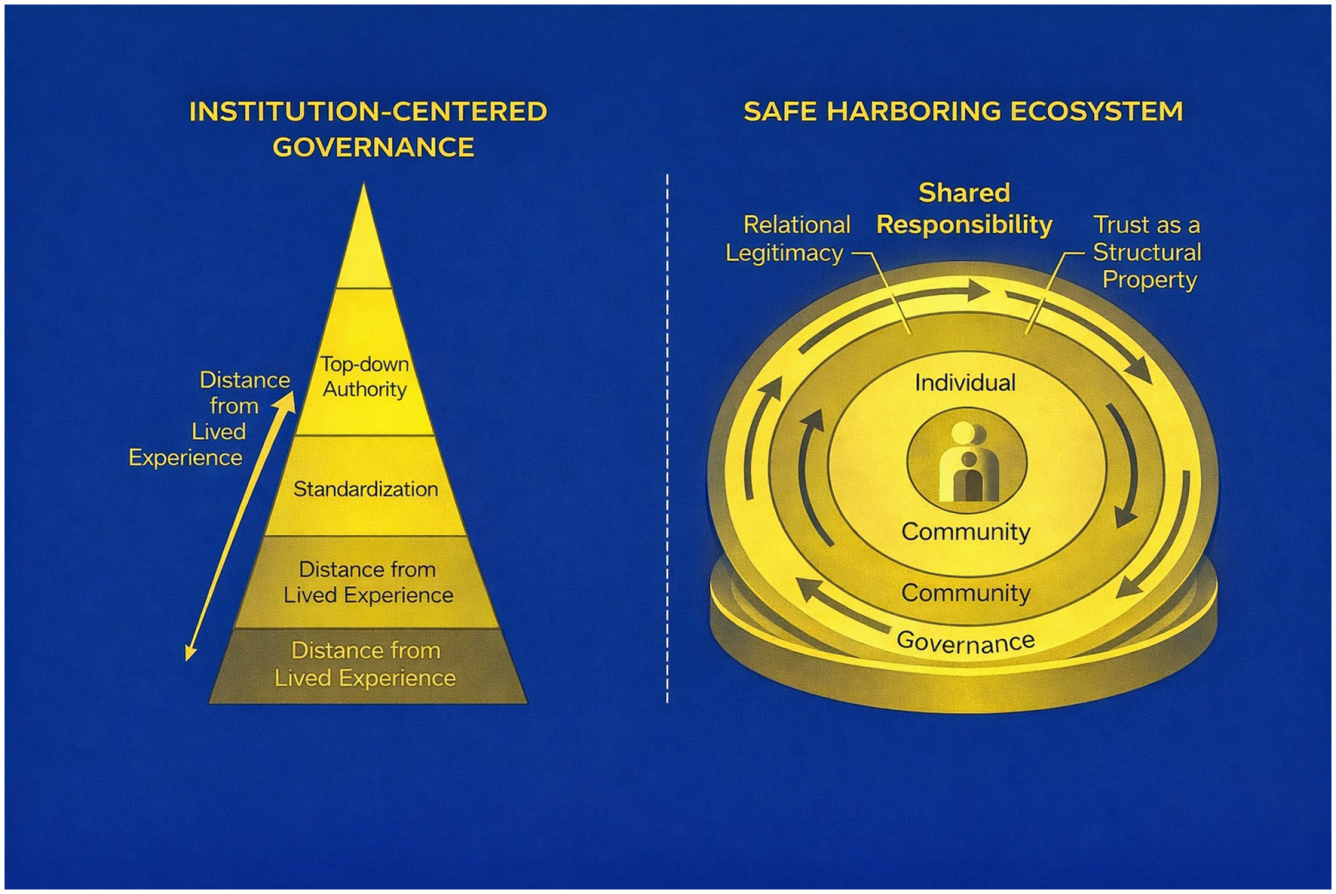
Governance design and safe harboring ecosystems. This figure contrasts institution-centered governance with a safe-harboring governance ecosystem. Institution-centered governance emphasizes hierarchy, standardization, and abstraction from lived experience. Safe harboring ecosystems embed individuals within layered relational contexts that distribute responsibility and sustain continuity. Trust and legitimacy emerge as structural properties of governance design rather than outcomes to be managed.

Within a *safe harboring* framework, trust and legitimacy emerge as structural properties of governance rather than as variables to be managed through discrete interventions. Continuity enables signals to be interpreted within context, distributed responsibility maintains identifiable accountability, and sustained participation supports relational legitimacy over time. Figure 2 contrasts these governance forms, illustrating how institutional design can either preserve or disrupt the relational conditions necessary for coherent, trustworthy care systems.

## Discussion

Taken together, these analyses support a single interpretive conclusion: that the capacity of health systems to integrate care is determined less by technical coordination than by the governance structures that organize responsibility, participation, and continuity over time. This Perspective integrates sociological theory with a relational governance model informed by the Sundance Ecosystem. The Sundance is not presented as empirical evidence or as a transferable Indigenous knowledge system. Rather, familiarity with Lakota relational practices serves as an orienting lens that clarifies governance features often obscured within institution-centered health systems, including continuity, distributed responsibility, and legitimacy grounded in sustained participation rather than formal role. This interpretive use is consistent with sociological approaches that examine how governance structures shape trust, accountability, and system coherence across contexts (Gilson, 2025; Peters, 2024).

In this sense, the Sundance informs the articulation of *safe harboring* as a governance model defined by its structural properties rather than its cultural origin. Its relevance lies in its capacity to illuminate how relational systems sustain participation, legitimacy, and trust over time—features that governance scholarship identifies as central to effective and trustworthy health systems (Kalulu et al., 2025; Menon et al., 2025). By contrast, institution-centered systems often rely on hierarchical control and standardized processes that, while necessary for coordination, may attenuate feedback, diffuse responsibility, and weaken relational accountability when scaled (Haby et al., 2025; Plsek and Greenhalgh, 2001).

Women's health serves as a structural stress test for governance arrangements in this analysis. Conditions such as Primary Ovarian Insufficiency require longitudinal interpretation across multiple physiological systems and life domains, placing sustained demands on continuity, coordination, and interpretive coherence (Nelson, 2009; Haggerty et al., 2003). Empirical research demonstrates that fragmentation in care is associated with coordination gaps and adverse outcomes, while continuity of care supports improved outcomes and reduced mortality (Kern et al., 2024; Pereira Gray et al., 2018). These findings suggest that fragmentation persists not because integration is conceptually unclear, but because governance arrangements often lack mechanisms for sustaining relational continuity, feedback, and responsibility across domains.

From this perspective, the contribution of *safe harboring* lies in reframing integration as a governance condition rather than a coordination problem. It highlights that the capacity of health systems to sustain continuity, legitimacy, and trust depends on how authority, responsibility, and participation are structured across institutional layers. In doing so, the framework offers a way to interpret persistent fragmentation in women's health not as a failure of knowledge or intent, but as a predictable outcome of governance design.

## Implications for governance in primary ovarian insufficiency

If *safe harboring* is understood as a governance condition rather than a conceptual abstraction, it becomes possible to consider how such an approach might be initiated in the context of Primary Ovarian Insufficiency. In clinical care, this would require a shift from coordinating episodic encounters toward establishing durable relational anchoring, in which women are supported by a stable longitudinal care structure capable of sustaining interpretation across endocrine, reproductive, cardiovascular, and psychosocial domains over time. Within such a configuration, responsibility for integrating information would remain within the care system rather than being displaced onto the individual navigating it, preserving identifiable accountability and reducing the burden of fragmentation. Similarly, digital and administrative infrastructures would need to be aligned with this relational logic, supporting continuity and shared interpretation rather than introducing abstraction or duplication. In human investigation, a safe harboring approach would require governance structures that counter institutional, temporal, and epistemic fragmentation by sustaining longitudinal engagement, integrating domains of expertise within a shared interpretive framework, and maintaining accountability to lived experience alongside clinical and biological data. These considerations do not represent a separate intervention layer but rather illustrate how governance design could be re-specified to sustain continuity, responsibility, and legitimacy across both care and research.

## Conclusion

This article posits that fragmentation in women's health research reflects the design of governance rather than inherent biological or demographic complexity. What appears to be a coordination problem may, more fundamentally, be a consequence of how authority, responsibility, and interpretation are structured within health systems. By reframing integration as a governance condition rather than a technical task, this Perspective highlights the need for relational infrastructures that sustain continuity, preserve legitimacy, and maintain identifiable responsibility across time.

*Safe harboring* offers a sociologically grounded governance model that bridges medical sociology, public health governance, and women's health research. Its contribution lies in clarifying how systems can remain accountable to lived experience while operating at scale. Treating women's health as a structural stress test makes visible the limits of institution-centered design and the necessity of governance arrangements that sustain relational continuity amid complexity. In this view, the capacity to maintain continuity, shared responsibility, and meaningful participation is not ancillary to effective care—it is the structural foundation upon which trustworthy and adaptive health systems depend.

## Data Availability

The original contributions presented in the study are included in the article/supplementary material, further inquiries can be directed to the corresponding author.

## References

[B1] Abdelaziz S. Garfield S. Neves A. L. Lloyd J. Norton J. van Dael J. . (2024). Unintended patient safety consequences of healthcare technologies. BMJ Open 14:e089026. doi: 10.1136/bmjopen-2024-089026 PMC11603710 39608995

[B2] Catches P. (1999). Sacred Fireplace (Oceti Wakan): Life and Teachings of a Lakota Medicine Man. Santa Fe, NM: Clear Light Publishers.

[B3] Deloria V. Jr. (1994). God is Red: A Native View of Religion. Golden, CO: Fulcrum Publishing.

[B4] Gilson L. (2025). Health systems as human systems: reflexivity, relationships, and resilience in the pursuit of the SDGs. Front. Public Health 13:1653839. doi: 10.3389/fpubh.2025.1653839 40910051 PMC12405280

[B5] Gone J. P. (2013). Redressing First Nations historical trauma: theorizing mechanisms for Indigenous culture as mental health treatment. Transcult. Psychiatry 50, 683–706. doi: 10.1177/1363461513487669 23715822

[B6] Greenhalgh T. Potts H. W. Wong G. Bark P. Swinglehurst D. (2009). Tensions and paradoxes in electronic patient record research: a systematic literature review using the meta-narrative method. Milbank Q. 87, 729–788. doi: 10.1111/j.1468-0009.2009.00578.x 20021585 PMC2888022

[B7] Haby M. M. Reveiz L. Thomas R. Jordan H. (2025). An integrated framework to guide evidence-informed public health policymaking. J. Public Health Policy 46, 193–210. doi: 10.1057/s41271-024-00535-9 39799247 PMC11893451

[B8] Haggerty J. L. Reid R. J. Freeman G. K. Starfield B. H. Adair C. E. McKendry R. . (2003). Continuity of care: a multidisciplinary review. BMJ 327, 1219–1221. doi: 10.1136/bmj.327.7425.1219 14630762 PMC274066

[B9] Kalulu P. Fisher A. Whitter G. Sener I. Doering M. Carter D. B. . (2025). Trust, trust repair, and public health: a scoping review. Front. Public Health 13:1560089. doi: 10.3389/fpubh.2025.1560089 40575096 PMC12199165

[B10] Kern L. M. Lau J. D. Rajan M. Rhodes J. D. Casalino L. P. Colantonio L. D. . (2024). Associations among claims-based care fragmentation, self-reported gaps in care coordination, and self-reported adverse events. BMC Health Serv. Res. 24:1045. doi: 10.1186/s12913-024-11440-y 39256705 PMC11389281

[B11] Kirmayer L. J. Dandeneau S. Marshall E. Phillips M. K. Williamson K. J. (2011). Rethinking resilience from indigenous perspectives. Can. J. Psychiatry 56, 84–91. doi: 10.1177/070674371105600203 21333035

[B12] Kleinman A. (1978). Concepts and a model for the comparison of medical systems as cultural systems. Soc. Sci. Med. 12, 85–95. doi: 10.1016/0160-7987(78)90014-5 358402

[B13] Kyle M. A. Feng K. Y. Wade C. G. Yaver M. (2025). Patient administrative burden: a scoping review. Health Aff. Sch. 3:qxaf216. doi: 10.1093/haschl/qxaf216 41278120 PMC12637203

[B14] Menon A. Kavanagh N. M. Falkenbach M. Wismar M. Greer S. L. (2025). The role of health and health systems in shaping political engagement and rebuilding trust in democratic institutions. Lancet Reg. Health Eur. 53:101326. doi: 10.1016/j.lanepe.2025.101326 40485753 PMC12144495

[B15] Nelson L. M. (2009). Clinical practice. Primary ovarian insufficiency. N. Engl. J. Med. 360, 606–614. doi: 10.1056/NEJMcp0808697 19196677 PMC2762081

[B16] Pereira Gray D. J. Sidaway-Lee K. White E. Thorne A. Evans P. H. (2018). Continuity of care with doctors—a matter of life and death? A systematic review of continuity of care and mortality. BMJ Open 8:e021161. doi: 10.1136/bmjopen-2017-021161 29959146 PMC6042583

[B17] Peters D. H. (2024). Building trust and trustworthiness in public institutions: essential elements in placing trust at the heart of health policy and systems. Int. J. Health Policy Manag. 13:8782. doi: 10.34172/ijhpm.8782 39620513 PMC11549569

[B18] Plsek P. E. Greenhalgh T. (2001). Complexity science: the challenge of complexity in health care. BMJ 323, 625–628. doi: 10.1136/bmj.323.7313.625 11557716 PMC1121189

[B19] Powers W. K. (1977). Oglala Religion. Lincoln: University of Nebraska Press.

[B20] Redvers N. Odugleh-Kolev A. Cordero J. P. Zerwas F. Zitoun N. M. Kamalabadi Y. M. . (2024). Relational community engagement within health interventions at varied outcome scales. PLoS Glob. Public Health 4:e0003193. doi: 10.1371/journal.pgph.0003193 38861576 PMC11166349

[B21] Saikat S. Seifeldin R. Zhang Y. Nweje M. Shivji S. Schmets G. . (2025). Governance for public health across health and allied sectors: a scoping review. BMJ Public Health 3:e003542. doi: 10.1136/bmjph-2025-003542 41211583 PMC12593447

[B22] Walker J. R. (1982). Lakota Society. Lincoln: University of Nebraska Press.

